# Patient expectations of and experiences with a suicide risk identification algorithm in clinical practice

**DOI:** 10.1186/s12888-022-04129-1

**Published:** 2022-07-23

**Authors:** Bobbi Jo H. Yarborough, Scott P. Stumbo, Jennifer L. Schneider, Julie E. Richards, Stephanie A. Hooker, Rebecca C. Rossom

**Affiliations:** 1 Kaiser Permanente Northwest Center for Health Research, 3800 N Interstate Ave, Portland, OR 97227 USA; 2 Kaiser Permanente Washington Health Research Institute, WA Seattle, USA; 3 Department of Health Systems and Population Health, University of Washington, WA Seattle, USA; 4 HealthPartners Institute, MN Minneapolis, USA

**Keywords:** Suicide prevention, Risk prediction models, Implementation, Qualitative, Patient

## Abstract

**Background:**

Suicide risk prediction models derived from electronic health records (EHR) and insurance claims are a novel innovation in suicide prevention but patient perspectives on their use have been understudied.

**Methods:**

In this qualitative study, between March and November 2020, 62 patients were interviewed from three health systems: one anticipating implementation of an EHR-derived suicide risk prediction model and two others piloting different implementation approaches. Site-tailored interview guides focused on patients’ perceptions of this technology, concerns, and preferences for and experiences with suicide risk prediction model implementation in clinical practice. A constant comparative analytic approach was used to derive themes.

**Results:**

Interview participants were generally supportive of suicide risk prediction models derived from EHR data. Concerns included apprehension about inducing anxiety and suicidal thoughts, or triggering coercive treatment, particularly among those who reported prior negative experiences seeking mental health care. Participants who were engaged in mental health care or case management expected to be asked about their suicide risk and largely appreciated suicide risk conversations, particularly by clinicians comfortable discussing suicidality.

**Conclusion:**

Most patients approved of suicide risk models that use EHR data to identify patients at-risk for suicide. As health systems proceed to implement such models, patient-centered care would involve dialogue initiated by clinicians experienced with assessing suicide risk during virtual or in person care encounters. Health systems should proactively monitor for negative consequences that result from risk model implementation to protect patient trust.

**Supplementary Information:**

The online version contains supplementary material available at 10.1186/s12888-022-04129-1.

## Background

Machine learning of electronic health records (EHR) and insurance claims data to predict risk of suicide attempt is a novel innovation in suicide prevention. Several research teams in varied settings have demonstrated that such models can predict risk of suicide attempt or death with 60–80% classification accuracy [1–11]. Far fewer have investigated patient perspectives on whether and how this technology ought to be used. When patients receiving inpatient psychiatric treatment (*n* = 102) completed anonymous questionnaires and reacted to three hypothetical vignettes exploring different approaches to introducing a predictive model-driven suicide prevention program, negative reactions and privacy concerns were rare [12]. However, focus groups and a survey of 1,357 members of a large integrated health system revealed that although patients hypothetically supported this use of their health data, they had reservations about how risk models might be implemented [13]. Privacy was a universal concern, there was a preference that only trusted clinicians should have access to suicide risk information derived from risk models, and patients were worried about the potential for negative consequences including strain on the clinician-patient relationship, risk conversations causing anxiety for patients, and stigma [13]. As suicide risk prediction models advance toward implementation in health systems, better understanding of patients’ concerns and preferences for their use will help to design patient-centered suicide prevention interventions that minimize the risk of iatrogenic harms resulting from use of risk models.

Previous studies [12, 13] measured participants’ responses to hypothetical implementation of suicide risk models. Conducted in three health systems, the present study afforded the opportunity to extend that work by further exploring patients’ perceptions of suicide risk model technology, concerns, implementation preferences, and, among a subset, actual experiences of suicide risk prediction models being implemented during a pilot study. At one replication site considering future implementation of a suicide risk prediction model, qualitative interviews with patients focused on perceived benefits and risks of using suicide risk prediction models to identify patients at high suicide risk and implementation preferences. Two other sites conducted small implementation pilots where suicide risk models were in use, affording the opportunity to additionally study patient experiences of two different implementation approaches.

## Methods

### Settings

The settings were three healthcare systems that provide medical and specialty mental health care and insurance to enrolled patient/member populations: Kaiser Permanente Northwest (KPNW) and Washington (KPWA) regions, each serving approximately 600,000–700,000 members in Oregon and Washington, and HealthPartners (HP), which provides care to 1.2 million patients and insurance coverage to 1.8 million members in Minnesota and western Wisconsin. In these settings, patients/members are primarily covered by commercial insurance, Medicare, or Medicaid and are demographically similar to the service area population.

At the time of this study, KPNW was considering future implementation of a suicide risk prediction model and KPWA and HP were engaged in small-scale pilot projects implementing the Mental Health Research Network’s validated suicide attempt prediction model. The model predicts risk of suicide attempt within 90 days following an outpatient visit in a mental health specialty clinic; predictors include demographic and clinical characteristics (e.g., psychiatric and substance use diagnoses and medications, elevated depression screening scores, history of self-harm or suicide attempt) [11].

At KPWA, the risk prediction model identified the top 5% at risk for suicide attempt or death. During the pilot, an electronic health record indicator flagged identified patients with an upcoming scheduled appointment at one outpatient mental health clinic. This flag was intended to prompt the clinician to assess suicide risk using the Columbia Suicide Severity Rating Scale (C-SSRS) [14] at the visit. C-SSRS administration was already part of a suicide prevention workflow for patients endorsing frequent suicidal ideation on the Patient Health Questionnaire (PHQ-9) [15]; however, clinicians were encouraged to administer the C-SSRS for patients identified by the risk model even if ideation was denied on the PHQ-9 or it was not administered.

At HP, the pilot implementation was administered through an existing case management program for insurance plan members who were high utilizers of outpatient care, had frequent psychiatric hospitalizations, or had ongoing serious mental illnesses or substance use disorders. Monthly, the suicide risk models were run, outreach telephone calls (efforts to reach out to patients at risk, hereafter referred to as outreach) were made to individuals identified in the top 2.5% at risk for suicide attempt or death, and case managers administered the C-SSRS. Use of the suicide risk model was not transparent to patients at either of the pilot implementation sites by design; at KPWA and HP use of the flag was intended to augment existing clinical workflows for assessing suicide risk and clinicians did not explicitly mention use of the risk models to their patients.

### Sample

A potential recruitment sample at KPNW was randomly selected from individuals identified through the EHR with either: 1) suicide ideation in the previous 12 months (based on their response to item 9 of the PHQ-9; 97%); or 2) evidence of a suicide attempt in the prior 12 months excluding the past 90 days (3%). These individuals were chosen to approximate the types of individuals who could be identified by suicide risk prediction models. At KPWA, patients identified by the suicide risk prediction model who had a subsequent mental health visit at the pilot implementation clinic during the study period were eligible for recruitment. At HP, individuals who were engaged in the care management program and identified by the risk prediction model during the study period were eligible once their telephone appointment with the care manager had taken place.

### Recruitment

All sites began recruitment by first mailing potential participants a letter explaining the study and an information sheet containing all elements of informed consent. The KPWA Institutional Review Board reviewed and approved all study materials and procedures. At KPNW, potential participants were randomly selected for the recruitment mailing from the randomly identified sample. For the two implementing sites, the letter was typically mailed 1–2 weeks following the visit or outreach call in which the risk conversation was to have taken place. The recruitment letters contained a local telephone number for participants to call to join the study or decline participation. Potential participants were called up to a maximum of six times.

Approximately three-quarters of the way into the recruitment period, two sites (KPNW, KPWA) adjusted their recruitment strategies because they were enrolling proportionally fewer males and persons of color. In order to hear from a broader selection of potential participants, both sites attempted to over-recruit males and persons of color to assure that we were capturing experiences of all individuals who had been or would potentially be identified by the use of suicide risk identification algorithms. We did this by randomly selecting and mailing recruitment letters to male and non-white individuals from our remaining recruitment pool as we had gender and race information from our electronic health records. Recruitment persisted with the goal of reaching saturation of interview themes (additional details below).

### Interviews

Three distinct but conceptually overlapping interview guides were customized to each setting; a consultant with implementation science expertise helped to refine the interview questions. Prior to conducting patient interviews, two community advisory boards, composed of members with lived experience of suicide or serious mental illness, reviewed all recruitment and interview materials and provided feedback to facilitate successful recruitment and interview completion.

At KPNW, questions were hypothetical in anticipation of future suicide risk model implementation and focused on acceptability of the use of suicide risk prediction models and patients’ expectations or preferences for their use. Interviews at KPWA and HP attempted to elicit patients’ experiences of risk conversations that occurred subsequent to identification by suicide risk models. One-on-one phone interviews, generally taken in participants’ homes, averaged approximately 45–60-min; they were conducted and audio-recorded using HIPAA-compliant software between March and November 2020 by a team of trained interviewers led by SPS and JS. Interviewers had broad expertise in qualitative methods and were primarily female, with master’s or doctoral training in psychology, public health, or sociology. Interviewers were unknown to participants, interviewer motivation for the research was not disclosed; interviewers were interested in suicide prevention, health system quality improvement, and implementation research. For quality assurance, transcripts were returned to interviewers to review, comment, and provide corrections as needed prior to coding. The KPWA Human Subjects Protection Office reviewed and approved all study materials; all participants provided informed consent.

### Analysis

We employed a constant comparative analysis approach [16–19] led by two trained qualitative methodologists (SPS, JS), and facilitated by Atlas.ti qualitative analysis software [20]. First, we reviewed six transcripts (two from each site) and any associated field notes to develop a preliminary codebook (See Additional file 1). We applied the resulting codes to those six transcripts and an additional four transcripts, meeting frequently to discuss coding progress, identify changes to the codebook (e.g., addition of new codes, clarifying definitions), and resolve coding discrepancies. Codes reflected interview questions (e.g., initial reaction to this method of risk identification; perceived benefits, value, harms, negative consequences) and content naturally emerging from the interview (e.g., experiences of stigma). We began the coding process while interviews were still being conducted. We identified one new topical area and created one new code and retroactively applied it to previously coded transcripts. We similarly made small changes in the definitions of codes and also retroactively applied those. By coding and interviewing simultaneously, and meeting regularly, we were able to determine that interviews conducted later produced less new information and more confirmatory information. We originally planned to conduct 15–20 interviews per site to capture the breadth of experiences and found that 20 per site was sufficient.

Following coding of the remaining transcripts, query reports for each code were created, read, and summarized by one of the two coding authors (SPS, JS). When coding was nearly complete, coders met with interviewers from all sites to debrief. This process sensitized the coders to be aware of certain topics from the different sites and served as a way of triangulating emerging themes [16, 21]. Next, the full analytic team (BJY, SS, JS) met weekly to discuss important themes and subthemes from each summarized query report, and how the results across all queries fit together as a whole. Results were derived from assessing the relative importance that patients attributed to critical aspects of suicide risk models generally and the implementation procedures more specifically. Thematic results were shared with interviewers from each site as a means of “member checking” interpretations [16–18].

## Results

Of the 146 patients invited to participate across all 3 sites, 62 completed interviews (43% response rate): 22 at KPNW; 20 at KPWA; and 20 at HP. Most potential participants passively refused (i.e., did not answer phone, did not return calls; 49%) rather than actively refusing (e.g., did not want to join due to topic sensitivity; 8%). We discontinued recruitment when we met our enrollment targets and completed interviews suggested we had reached saturation. Basic demographic characteristics of the individuals we interviewed are described in Table 1. Our efforts to balance our sample succeeded in reducing some of the identified demographic gaps but our final recruitment sample had slightly more participants identifying as female or Asian and less identifying as Black than our overall recruitment pool.

**Table 1:** Characteristics of recruitment sample

Characteristic	Interviewed^a^ (*n* = 62)		Not interviewed (*n* = 84)	
	n	%	n	%
Gender				
Female	36	58	46	55
Male	20	32	38	45
Nonbinary	6	10	^b^	^b^
Ethnicity				
Hispanic	6	10	4	5
Not Hispanic	55	89	43	51
Unknown	1	2	37	44
Race				
White	44	71	56	67
African American	2	3	10	12
Asian American	6	10	2	2
Native American/Alaska Native	2	3	2	2
Native Hawaiian/Pacific Islander	1	2	0	0
Multiple races reported	3	5	1	1
Other race reported	3	5	1	1
Unknown	1	2	12	14
Age categories				
18–24	17	28	24	29
25–34	18	29	26	31
35–44	11	18	16	19
45–54	9	15	8	10
55 or older	7	11	10	12

^a^All demographic data from completed interviews is based on self-report. Demographic data from non-interviewed individuals comes from the electronic health record. Not all numbers will add to 100% due to rounding

^b^Not available in the electronic health record

Thematic findings from our content analysis are organized by the following topical areas: general acceptability and reaction to risk prediction models, concerns about potential negative consequences, clinical experiences with actual risk prediction model implementation, and patient preferences and recommendations for implementation of suicide risk prediction models.

### EHR and claims-based suicide risk prediction models were generally acceptable

Most patients viewed the suicide risk models similarly to other health risk prediction models and found them acceptable. However, across all three sites, patients were aware of data limitations, and this caused some patients to feel ambivalent about use of the models. Two-thirds of patients (42 of 62) expressed a positive initial reaction to suicide risk prediction based on machine learning of EHR and claims data.

*“I think if there is a way to help screen or prevent somebody from dying or attempting suicide that it’s definitely worth maybe some minor… personal information and stuff that would be used…Since it’s not outside of a medical field, then it’s not really a huge…invasion of privacy.” (KPNW)*

Patients who were supportive of this innovation expressed opinions ranging from assumptions that health systems were already using EHR and insurance claims data in this manner, or that it was a benign use of in-hand data, to strong convictions that suicide risk prevention was a moral imperative and therefore risk prediction models were justified. Interviews deliberately explored whether participants viewed the risk factors used to predict suicide risk (e.g., mental health diagnoses, medications, previous suicide attempts) differently or more sensitively than those used in prediction for other health risks, for example cancer (e.g., smoking status) or heart disease risk identification (e.g., blood pressure). Many participants felt there should be no difference:

*“I’m sure some people would see it as like an invasion of privacy, just because it is very personal. But I personally feel like mental health and mental illnesses are medical. They’re just the same as somebody’s smoking habits and stuff like… a cancer diagnosis…and diabetes…it’s all medical data. Like you’re diagnosed with something, it should be just the same as being diagnosed with a mental illness (HP)*

Those who saw differences between prediction of suicide and other health risks perceived predictors of suicide ideation or intent as more variable than other health conditions:

*“I think that smoking is known to cause cancer and heart disease. And, I think mental illness is a lot harder to forecast” (KPWA)*

Patients understood the predictors that would likely influence suicide risk model scores. They also understood that important data relevant to suicide ideation and potential behaviors are not included in EHR or insurance claims datasets. Patients wondered if suicide risk models could capture, for example, the complex experiences of long-term chronic pain. Family history of suicide and substance use were also mentioned as important risk factors for suicide.

*“I: Are there other things you think would be important to consider, or that maybe need to be thought about in terms of what might need to go into that tool to be accurate?*

*R: Yeah. I think family history…of mental health issues, suicide. Alcohol consumption would be huge. Access to firearms for males.” (KPNW)*

A few patients expressed concerns about the accuracy of the data and that some people may not be willing to disclose important information due to fears it could lead to unwanted types of treatment.

*“It’s just a matter of having the correct data and making sure that you have all the right data. And also knowing the fact that some people…might not share every single thing about their risk factorization because they might be afraid that they would get hospitalized because of something that they would share” (HP)*

About a third of participants were ambivalent (*n* = 12) or unsupportive (*n* = 8) of suicide risk prediction models. Ambivalence often meant weighing the importance of suicide prevention with concerns about how the model could be misused. For example, one participant described how potential over-reaction could be unhelpful:

*“The first thought is kind of invasive…predetermining something that might not happen can be like too much involvement. On the other hand, secondary to that would be if it can help and not hinder then it is probably beneficial.” (HP)*

Another described how the model could result in denied access to healthcare:

*“It scares me a little bit. I tried to get life insurance. And you know my fear is that being in a capitalist world, people will use this information to deny service…But at the same time, I like that there’s an interest in protecting human beings. [But] you don’t know the potential negative impact it might have… (KPWA)*

### Patients were concerned about negative consequences

Data from all three sites suggested patients shared concerns about negative consequences. One concern that was not prompted by the interview guide but that patients spontaneously described often enough to warrant mention (*n* = 13) was that reaching out to individuals could induce anxiety or suicidal thoughts. *“I feel like I might end up focusing on that too much. And then that would end up in me having a higher risk of suicide.” (KPWA).*

Patients also described how, for some individuals, bringing up a discussion centered on suicide could worsen the mental health of the very people the health system was trying to protect.

*“.…they might deny everything and really, you know, panic and freak out and then cause their condition to worsen.” (HP)*

*“…if you’re not ready to hear that information…the approach could be detrimental… And if you approach them, they’re either…they’re gonna do one of two things. They’re going to resist or they’re going to accept. And it could go either way. That’s the problem.” (KPNW)*

A separate set of patients (only one participant in common with the 13 described above) were concerned about how identification by suicide risk models might escalate concerns and lead to coercive or inappropriate treatment or legal consequences (*n* = 13). Some reported that they had previously experienced involuntary psychiatric hospitalization or had police conduct a welfare check at their house; these experiences made them wary of suicide risk prediction models.

*“But my first thing is always what are you going to do to me? Meaning, are you going to try to hold me, you know like put me under a hold? Am I going to be sent into the tiny room and forced to sit there and, you know, be looked at? Is someone going to, you know, decide whether or not I can go home? So first of all, it would just strike fear in me. That’s the first thing that comes to mind.” (KPNW)*

### Patients receiving mental health services expected to be asked about their suicide risk (data from two implementing sites)

Use of the suicide risk model was not transparent to patients at either of the two implementing sites, and interview participants from KPWA and HP were usually unaware that they had been identified as high risk by this method. As such, interviewers could not ask directly about participants’ experiences being identified by suicide risk prediction models and instead sought to elicit memories and feelings about a recent visit or a recent call with a case manager where suicide may have been discussed. Participants responded generally about their recollections of the risk conversation (if they recalled having a risk conversation; some did not) rather than the method of identification and later were asked explicitly about their general feelings toward suicide risk prediction models.

At KPWA, the majority of patients (16 of 20) reported that they typically filled out depression screening questionnaires at each visit and expected to have conversations about suicide risk during mental health visits. Such discussions during recent visits did not seem out of context for most patients, particularly as these visits were typically with mental health clinicians with whom they had established relationships. A few individuals mentioned that initially they either didn’t want to talk about suicide ideation or felt it was unnecessary as they were not having those thoughts at the moment.

*“At first I was a little bit off-put just because I didn’t really expect to talk about that or open up about it. But after a while my care provider made me feel really comfortable with it...she was really understanding and she made me feel comfortable with the whole thing. And coming from someone that’s never really opened up a lot, it actually did help me a lot. So in the end I was actually okay with it.” (KPWA).*

At HP, most patients (at least 14 of 20) reported that they had an established relationship with the case manager and thus had some context for the assessment. Only three individuals specifically mentioned that the case manager and the outreach approach were new to them; one of those individuals reported feeling surprised at having a conversation about suicide when they were not expecting it but that it was generally acceptable. Another reported that the outreach did not seem timely as they were not having thoughts of suicide at that moment. More broadly, patients recalled having a conversation that included suicide risk screening and felt that was acceptable. Patients understood that clinicians were doing their job when they assessed for suicide risk. Very few patients reported feeling anxious about the discussion and none reported any deleterious effect on the relationship with the provider or the case manager.

*“[Recent outreach call] was basically, ‘I saw that you were in the hospital. How are you doing? Is there anything that I can provide for you?’ That kind of thing…And it could very well have just been me, because I was just pretty much out of the hospital and was not very talkative…. It was okay. It wasn’t super helpful….But it was supportive” (HP)*

### Patients strongly preferred that suicide risk communication come from a mental health professional with specific training in suicide assessment

Across all three sites, patients strongly preferred that risk communication come from a counselor/therapist with whom they had an established relationship; a minority were comfortable with their primary care clinician initiating a risk conversation with them, but only when there was a strong clinical relationship: *“I would prefer my therapist because they know a lot more about my situation than my psychiatrist and primary care provider do. And I trust them more too.” (HP).*

Most importantly, it was important to patients that the clinician have significant training in assessing and responding to suicide ideation.

*“I think that people need to be trained on how to have those kinds of conversations. I’ve had a couple instances where people clearly weren’t very knowledgeable about how to talk to someone with SI [suicide ideation]. It’s not inherently something that’s natural, even if you’re a health care provider, even if you’re in the mental health field*.” *(KPWA)*

Regarding the mode of communication with patients, participants noted strengths and gaps in the two main approaches—visit-based or outreach—studied here. Distinct advantages to implementing a suicide risk identification program during a mental health visit included that the approach was more personal or more natural (compared to an outreach call), that communication could be achieved through body language as well as verbally, that there was more context for such a discussion, and that some patients might be more willing to open up during a visit.

*“I personally would prefer face to face communications about those kind of things because you can obtain a lot of information about somebody when you meet them face to face. We convey so much body language...And I think, especially if it were a clinician or someone who was trained to give this information and then also to read the response of the recipient of the information, I think you’re going to get even more information that way.” (KPNW)*

However, some patients acknowledged that if a person were identified at-risk, passively waiting for them to make an appointment might be harmful; thus, a more proactive approach may be necessary. A majority of patients (50 of 62) found outreach to patients at risk acceptable. Patients felt an outreach approach demonstrated caring and concern by the health care system.

*“Just knowing somebody out there cares can go a long way. That there’s actually somebody who is calling to see how I’m doing. You know, even if it’s a short conversation it still gets in your head. And if the person has a very caring attitude and not a judgmental attitude, then I would think that that would help the person. I mean, even with my care manager just reaching out meant a lot. You know, she actually cares, you know?” (HP)*

When asked about email outreach, some patients appreciated that email seemed more private, that it would allow the patient time to collect their thoughts versus having a discussion in the moment, and that it would give the patient control over when and how to respond.

*“I think I would prefer an e-mail because that would give me time to think. You know, I wouldn’t be put on the spot because I wouldn’t be on the phone. It would make me a lot more comfortable. I would have a better chance of getting the care I needed if I needed it hearing it through an e-mail first rather than a phone call” (KPNW)*

Overall, patients recommended that a variety of approaches be used in suicide prevention. For example, participants suggested that if an at-risk patient did not come in for a visit, they should receive some form of outreach. If they did not answer their phone, they should receive a follow-up email. Finally, participants suggested if patients cannot be reached by these methods, the health system should send a caring letter inviting them to call in to report how they are doing.

## Discussion

In this qualitative study we sought to understand patients’ perceptions of suicide risk prediction models, concerns or preferences for implementation, and, among a subset, clinical experiences where suicide risk prediction models had been implemented and patients recalled having a suicide risk discussion.

Most participants favored risk prediction using machine learning of EHRs. Those who were initially ambivalent could see advantages and disadvantages of this technology. Many people who were reluctant described negative personal experiences with mental health care (particularly coercive treatment) that motivated their responses. Some individuals were concerned about negative consequences. Some participants voiced a common misconception that such conversations may trigger or worsen someone’s mental health condition or induce suicidal thoughts. Given that a review of the published literature examining whether asking about suicide induces suicidal ideation found that acknowledging and talking about suicide may reduce, rather than increase, suicidal ideation, and may lead to improvements in mental health [22], concern about exacerbating suicidal ideation should not prevent use of this technology. Further, though interviewers did not explicitly explore this concern among those who had experienced risk identification by these methods, there was no evidence that participants were distressed in this manner; while some were a bit surprised by outreach calls checking in about suicidal ideation, none reported worsening of mental health or spontaneous suicidal ideation as a result.

Some participants were concerned that outreach to patients who did not self-identify as at-risk for suicide could be unwelcomed. Again, among people who experienced outreach calls, most appreciated the outreach, even when the timing of the call did not occur when patients identified as at-risk. Many felt the approach was caring and respectful. However, most, if not all, of these patients were unaware that they had been identified as high risk by a suicide risk prediction algorithm. Some patients were concerned being identified as high-risk could result in coercive treatment, like involuntary psychiatric hospitalization. Future research on this topic should consider measuring health care utilization, including psychiatric hospitalizations, following implementation of suicide risk prediction algorithms.

These findings are consistent with the few previous studies that have assessed patient perspectives of suicide risk identification through risk models developed using health records [12, 13], and suicide risk assessment more generally [23–25]. Patients generally support implementation of suicide risk prediction models, but also report fear of coercive treatment or stigma resulting from identification. Patients in prior studies designed to elicit experiences with suicide risk assessment [23–25] have also expressed ambivalence about identification and described weighing hope for help with fears of negative consequences and misuse of reported suicidality. The present study extends these findings to include patient preferences for implementation, which have clinical and logistical implications for organizing this type of care. Patients strongly prefer suicide risk communication come from a mental health professional with specific suicide prevention training. Health systems considering implementation of suicide risk prediction models need to plan resources such that access to suicide prevention specialists is available for patients identified at risk. Negative or stigmatizing experiences with health care providers may lead to avoidance of health services, crisis, and involuntary services [26]. Visit-based risk conversations, phone and email outreach were all acceptable. Patients endorsed a stepped-outreach approach and felt such efforts would demonstrate care by the health system.

A few limitations should be noted. Two of the three health systems have robust suicide prevention programs in place, so risk assessment and follow up is normalized in these settings and patient reactions may not generalize to health systems with less vigorous screening in place. One of the health systems was not an implementation site so patients were responding to hypothetical scenarios, however their responses were consistent with those by patients in the pilot implementation settings. While we attempted to balance our interview sample on various demographic characteristics, it may not completely represent the targeted population. For example, our sampling approach resulted in a slight over-representation of participants identifying as female or Asian and an under-representation of Black perspectives (compared to the proportions available to be interviewed in the recruitment sample). Gender- or race-based experiences may influence acceptability of suicide risk model use. For example, it is known that risk models predicting suicide death (a rarer event compared to suicide attempt) may exacerbate health disparities, distrust of suicide risk models would certainly be expected to influence their acceptability. In spite of these relative methodological weaknesses, these results are important given the paucity of data on patient perspectives on use of this technology for suicide prevention.

## Conclusion

Patients generally approved of suicide risk prediction models that use EHR data to identify patients at-risk for suicide. As health systems proceed to implement such models, patient-centered care would involve dialogue initiated by trained clinicians experienced with assessing suicide risk virtually or in office visits. Health systems should proactively monitor for negative consequences that result from risk model implementation, to protect patient trust.

## Supplementary Information

**Additional file 1: Appendix 1.** Patient expectations of and experiences with a suicide risk identification algorithm in clinical practice. Accompanying codebook used in qualitative analysis.

## Data Availability

The datasets generated or analyzed during the current study are not publicly available because asking participants to consent to interview transcripts or a publicly shared dataset derived from interview content could compromise the quality of the research—the requirement for data sharing could influence the types of individuals willing to participate in the study or the type of information participants are willing to share. Further, maintaining our study participants’ confidentiality is of paramount importance. It may not be possible to sufficiently anonymize or redact transcripts to prevent the possibility of deductive disclosure. Our qualitative interview guides are freely available to any interested researchers. We will also provide full code books upon request. Requests for interview guides or code books should be sent to Dr. Yarborough.

## Abbreviations

C-SSRS: Columbia Suicide Severity Rating Scale
EHR: Electronic health record
HP: HealthPartners
KPNW: Kaiser Permanente Northwest
KPWA: Kaiser Permanente Washington
PHQ-9: Patient Health Questionnaire
