# Supplementary material for: Patient expectations of and experiences with a suicide risk identification algorithm in clinical practice
Source: BMC Psychiatry. 2022 Jul 23;22:494. doi: 10.1186/s12888-022-04129-1 (PMC9308306; doi:10.1186/s12888-022-04129-1)
Supplement: Supplementary file 1 — Additional file 1: Appendix 1. Patient expectations of and experiences with a suicide risk identification algorithm in clinical practice. Accompanying codebook used in qualitative analysis. [file 12888_2022_4129_MOESM1_ESM.pdf]

Appendix 1. Patient expectations of and experiences with a suicide risk identification algorithm in clinical practice. Accompanying codebook used in qualitative analysis

**Code**	**Definition**
Barriers	Patient perceived barriers to implementing or using this type of identification process; could include concerns about accuracy, lack of available resources, lack of concern from the health care system, privacy concerns, the complex or subjective nature of topic (e.g. each person is so unique)
Benefits	Benefits that could come from using a risk identification program, e.g. preventing suicide, education, making someone more self-aware; earlier outreach from health system
Changed opinion	Participant reflections on whether or not their opinion or feelings about the tool have changed or not after discussing it in more depth. Can include comments about usefulness or not, comfort level or not, etc.
Communication preferences	Discussions of how to start a conversation on suicide, what type of approach to use (e.g., start gently); what communication should follow from an identification; overall messaging and tone.
Disagreement/disconnect	The difference between the possibility of being identified as at risk but not feeling that way; reactions to how that would feel including conversations with doctor about that; could include feelings of how this process feels impersonal
Family involvement	Feelings about whether family should or should not be involved or notified
Harms/negative consequences	Any expression of concern that this type of identification could result in what the individual perceives as a harm. Harms could include over-reactions by the health care system (forced hospitalization or medication), stigma in future health care, disclosure to individuals who should not know, disclosure outside the health system (fears for future insurance), create worry about suicide risk when don't feel this way. Could also include "intrusiveness" in one's personal life or invasion of privacy
Implementation (at two implementing sites)	Any discussion of what the patient perceives about the implementation at that site (or whether they know anything about it); includes not knowing that the implementation is happening or if it feels the "same" like usual standard of care (e.g. not really any different than in past) or different than in past
Initial reaction to tool	Participant response to initial reaction question, can include comfort or "I'm okay, good idea" or more hesitancy in reaction like "I'm not sure, it depends"
Mode--outreach call	Discussion of how participants feel about receiving a call as the mode of outreach to discuss risk identification – also includes preferences on who communication should come from (e.g PCP, therapist or other)
Mode--email	Discussion of how participants feel about receiving an email (or other electronic health record notification) as the mode of outreach to discuss risk identification - – also includes

	preferences on who communication should come from (e.g PCP, therapist or other)
Mode--next appointment	Discussion of how participants feel about waiting until their next scheduled appointment as the mode of discussing risk identification - – also includes preferences on who communication should come from (e.g PCP, therapist or other)
Mode--favorite/preferred	Participant reports of what they would prefer as their favorite mode of communication to receive risk identification information - – also includes preferences on "who" communication should come from (e.g PCP, therapist or other).
Next steps & advice	Discussions of how the tool should be used in implementation; patient preferences for how it will work; discussion or resources or activities that could/may or do (KPWA/HP) result from a risk identification implementation; this includes the who (e.g., prefer therapist) and the how of what happens next. Could include discussions of patient choices and options, shared-decision making; and any advice or recommendations patients' have regarding implementation of the tool<br><br>Also includes discussions of "well I'm not sure how I feel about it because it's so dependent on how it is used"
Patient-provider relationship	Discussions about how the relationship between patient and provider (primary care or mental health) could impact how tool is utilized or how patient may respond to outreach from the tool. Could include issues of trust and communication and may at times overlap with "disagreement/disconnect" and/or "communication preferences" codes.
Patient story	Patient story about their life with mental illness or previous interactions with health system about MH issues, or suicide-related experiences. Could also include discussions of current state, or impact by COVID etc.
Reactions to data	Could include discussions of the sensitive nature of the data used in algorithms, the type of data that is potentially included in the algorithm, reactions to the concept of using data in this way (algorithm) or how it compares to use of data for other tools like heart disease; could be about the accuracy of the data; how would the algorithm find me (e.g., what went into it? I'd want to know what went into it)<br><br>Includes how MH data don't seem that different than other types of data ; Also if patients are generally "okay" with this type of data or pose questions about it.
Responsibility & ethics	Health system responsibility to identify and take action to prevent suicide; ethics of how that happens and who could be harmed or left out; may also include patient's questioning what is meant by "responsibility" or their hesitancy around this

Appendix 1. Patient expectations of and experiences with a suicide risk identification algorithm in clinical practice. Accompanying codebook used in qualitative analysis
